# P-325. An Innovative Approach For An HIV-1 Prophylactic Vaccine – Results From A Phase I Clinical Trial

**DOI:** 10.1093/ofid/ofaf695.544

**Published:** 2026-01-11

**Authors:** Pedro Garbes, Christina Chang, Howard Wright, Ryan Norton, Stacey Ouyang, Shaunna Shen, Hongmei Gao, David C Montefiori, Jack Heptinstall, Sheetal Sawant, Georgia Tomaras, Jiang Zhu, Keegan Braz Gomes, Mary Giffear, Kevin O’Neill

**Affiliations:** Uvax Bio, Weston, MA; Nucleus Network, Melbourne, Victoria, Australia; Avance Clinical, Melbourne, Victoria, Australia; Avance Clinical, Melbourne, Victoria, Australia; Avance Clinical, Melbourne, Victoria, Australia; Duke University, Durham, North Carolina; Duke University, Durham, North Carolina; Duke University Medical Center, Durham, North Carolina; Duke University, Durham, North Carolina; Duke University, Durham, North Carolina; Duke University, Durham, North Carolina; Scripps Research, La Jolla, California; Uvax Bio, Weston, MA; Uvax Bio, Weston, MA; Uvax Bio, Weston, MA

## Abstract

**Background:**

These are 1c-SApNP vaccines displaying HIV Env trimers. One of the candidates had its glycans trimmed while the other didn't. This innovative approach was recognized by IUPAC among the Top 10 Emerging Technologies.

**Figure 1.**
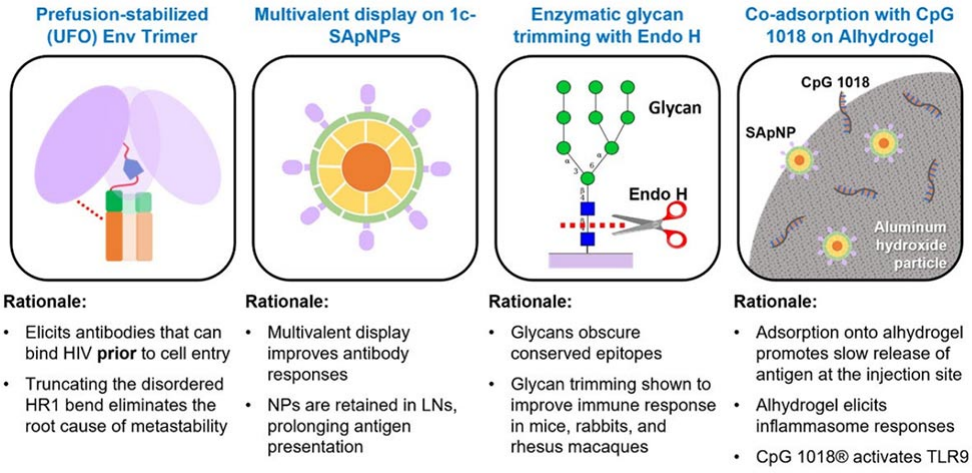
Vaccine Strategy Overview of Vaccine Candidates Design : UVAX-1197 and UVAX-1107

**Figure 2.**
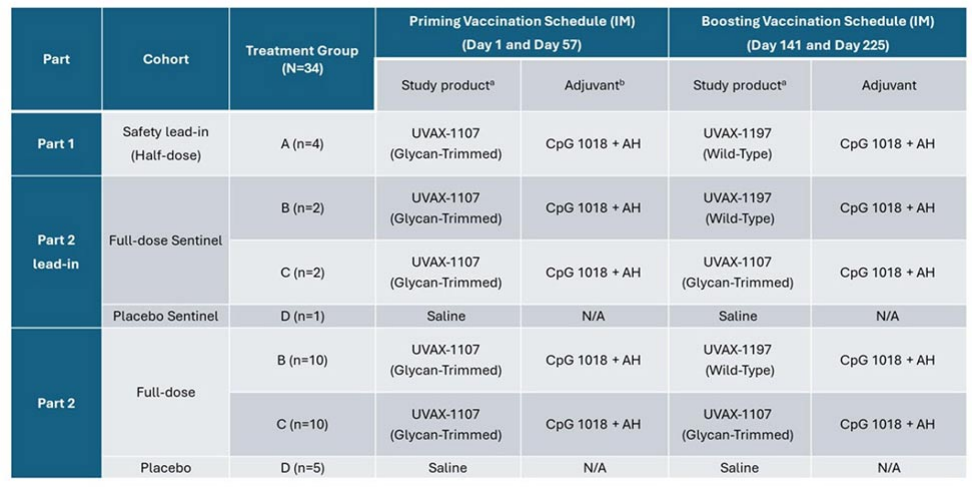
HIV-101 Study Randomization Scheme and Dosing Schedule

Abbreviations: AH, aluminum hydroxide; CpG 1018® cytosine phosphoguanine (CpG) adjuvant, N/A, not applicable.

a Doses of UVAX-1107 and UVAX-1197 were ∼165 µg for Part 1 and ∼330 µg for Part 2 doses.

**Methods:**

NCT06541093 is an HIV-1 vaccine adjuvanted (CpG1018/AH) trial. Vaccines design/study scheme are shown in Fig.1/2. Part 1 enrolled 4 participants receiving half-dose of 1107 followed by half-dose boost of 1197. Safety was reviewed after Part 1 participants reached D8, before opening Part 2. In Part 2, participants received a full dose of 1107 followed by 2 boosts with either full-dose 1107 or 1197. Safety was reviewed after the first 5 Part 2 participants reached D8, before randomization of the remaining population. Primary objective: To assess reactogenicity and to determine anti-Env IgG responses 2 weeks following each dose. Other outcome measures are shown in fig.3. We will present full results from the safety and per-protocol populations.

**Figure 3.**
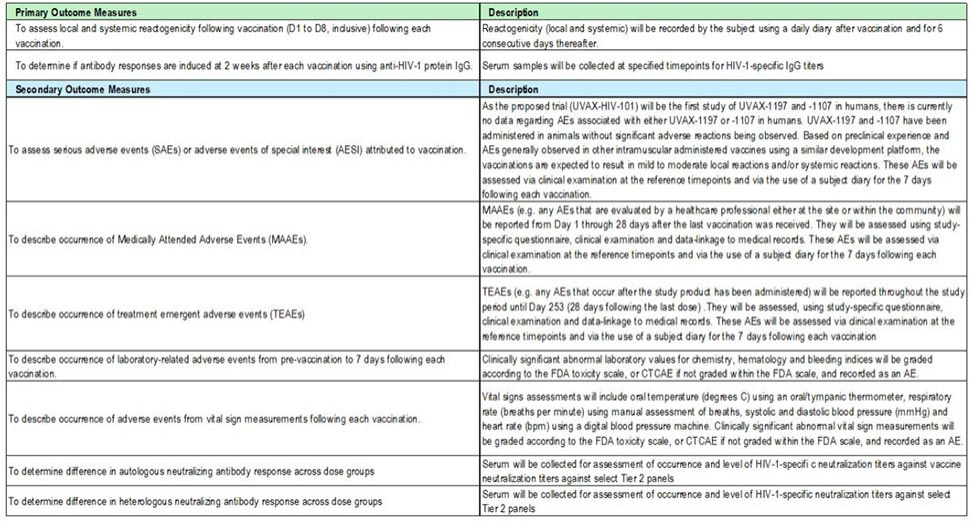
NCT06541093 Outcome Measures

Local and Systemic Reactogenicity Adverse Events in NCT06541093 clinical trial (Safety Population).

**Figure:**
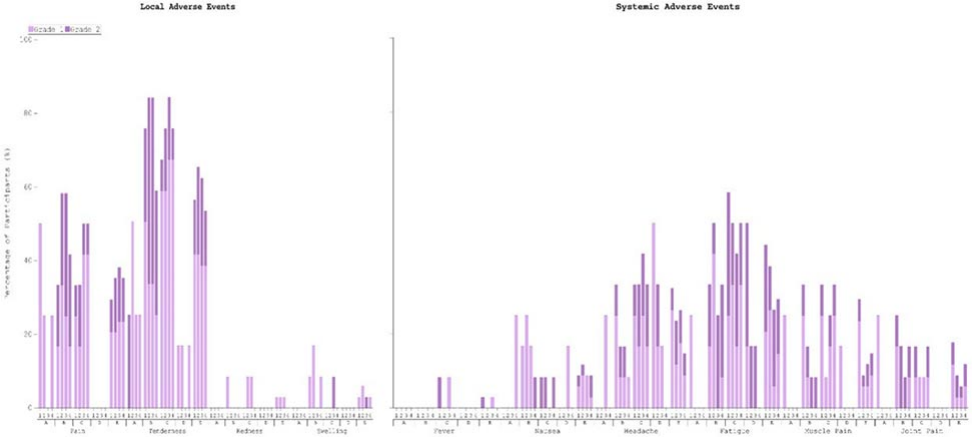

A=Part 1, Cohort Safety lead-In (Half-Dose), Treatment Group A; B=Priming Vaccination with Full-Dose UVAX-1107, followed by Boosting Vaccination with Full-Dose UVAX-1197; C= Priming Vaccination with Full-Dose UVAX-1107, followed by Boosting Vaccination with Full-Dose UVAX-1107; D=Pooled Placebo; E=Overall (A, B, C, D combined).

1=1st Priming Vaccination (1st vaccination/dose); 2=2nd Priming Vaccination (2nd vaccination/dose); 3=1st Boosting Vaccination (3rd vaccination/dose); 4=2nd Boosting Vaccination (4th vaccination/dose).

**Results:**

34 healthy participants were enrolled (balanced gender/race). Reactogenicity was mostly mild with few reports of moderate intensity. There was only 1 event of tenderness and fatigue that lasted more than 7 days after boost. Reactogenicity after doses are summarized in Fig.4. No vaccine-related SAEs were reported. Lab abnormalities were non-clinically significant, mild-to-moderate, and self-limited; a non-related fatal SAE occurred in a participant after the 1^st^ dose and was evaluated by the IDMC; a case of moderate Bell’s Palsy temporally related to the 3^rd^ dose was reported, and the participant fully recovered after 6 months. IgG responses were seen 14 days after the 1^st^ dose, peaking 14 days after 2^nd^, and remained elevated post-3^rd^ dose. Tier 1 neutralization was observed post-2^nd^ dose. Full antibodies kinetics (both binding IgGs and neutralizing antibodies) will be presented.

**Conclusion:**

1. UVAX-1107/1197 was well-tolerated.

2. These vaccines induced: a. Strong IgG responses against autologous antigens. b. A modest tier 1 NAb response. c. An autologous tier 2 NAb response in a subset of participants (this is the first report of autologous tier 2 neutralization for an HIV-1 vaccine using CpG 1018^®^/AH).

3. This innovative vaccine design/schedule/adjuvants showed potential for eliciting tier 2 neutralization.

**Disclosures:**

Pedro Garbes, MD, Uvax Bio: Honoraria Christina Chang, MBBS, Nucleus Network: Honoraria Howard Wright, MBBS, Avance Clinical: Advisor/Consultant Ryan Norton, BS, Avance Clinical: Honoraria Stacey Ouyang, MS, Avance Clinical: Honoraria Shaunna Shen, PhD, Duke University: Honoraria Hongmei Gao, MD, Duke University: Honoraria David C. Montefiori, PhD, Duke University: Honoraria Jack Heptinstall, BS, Duke University: Honoraria Sheetal Sawant, MPH, Duke University: Honoraria Georgia Tomaras, PhD, Duke University: Honoraria Jiang Zhu, PhD, Scripps Research: Honoraria|Uvax Bio: Advisor/Consultant|Uvax Bio: Board Member Keegan Braz Gomes, PhD, Uvax Bio: Honoraria Mary Giffear, BS, Uvax Bio: Honoraria Kevin O'Neill, BS, Uvax Bio: Honoraria

